# Supplementary material for: Beneficial Effects of Essential Oils from the Mediterranean Diet on Gut Microbiota and Their Metabolites in Ischemic Heart Disease and Type-2 Diabetes Mellitus
Source: Nutrients. 2022 Nov 3;14(21):4650. doi: 10.3390/nu14214650 (PMC9657080; doi:10.3390/nu14214650)
Supplement: Supplementary file 1 [file nutrients-14-04650-s001.zip › Figure S1.pptx]

## Slide 1
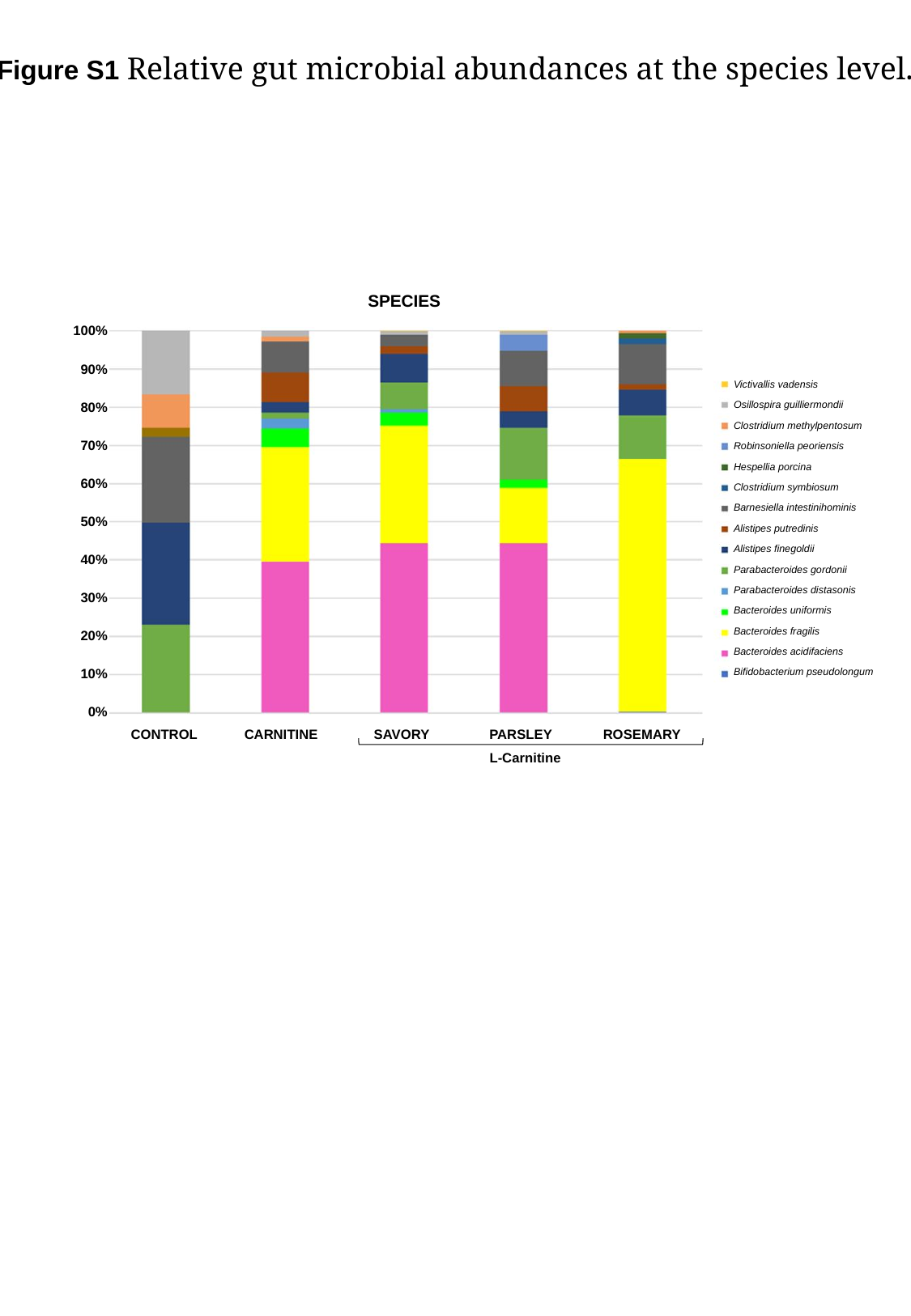

Figure S1 Relative gut microbial abundances at the species level.
SPECIES
100%
90%
80%
70%
60%
50%
40%
30%
20%
10%
 0%
Victivallis vadensis
Osillospira guilliermondii
Clostridium methylpentosum
Robinsoniella peoriensis
Hespellia porcina
Clostridium symbiosum
Barnesiella intestinihominis
Alistipes putredinis
Alistipes finegoldii
Parabacteroides gordonii
Parabacteroides distasonis
Bacteroides uniformis
Bacteroides fragilis
Bacteroides acidifaciens
Bifidobacterium pseudolongum
ROSEMARY
CONTROL
CARNITINE
PARSLEY
SAVORY
L-Carnitine
