# Supplementary material for: Beneficial Effects of Essential Oils from the Mediterranean Diet on Gut Microbiota and Their Metabolites in Ischemic Heart Disease and Type-2 Diabetes Mellitus
Source: Nutrients. 2022 Nov 3;14(21):4650. doi: 10.3390/nu14214650 (PMC9657080; doi:10.3390/nu14214650)
Supplement: Supplementary file 1 [file nutrients-14-04650-s001.zip › Figure S2.pptx]

## Slide 1
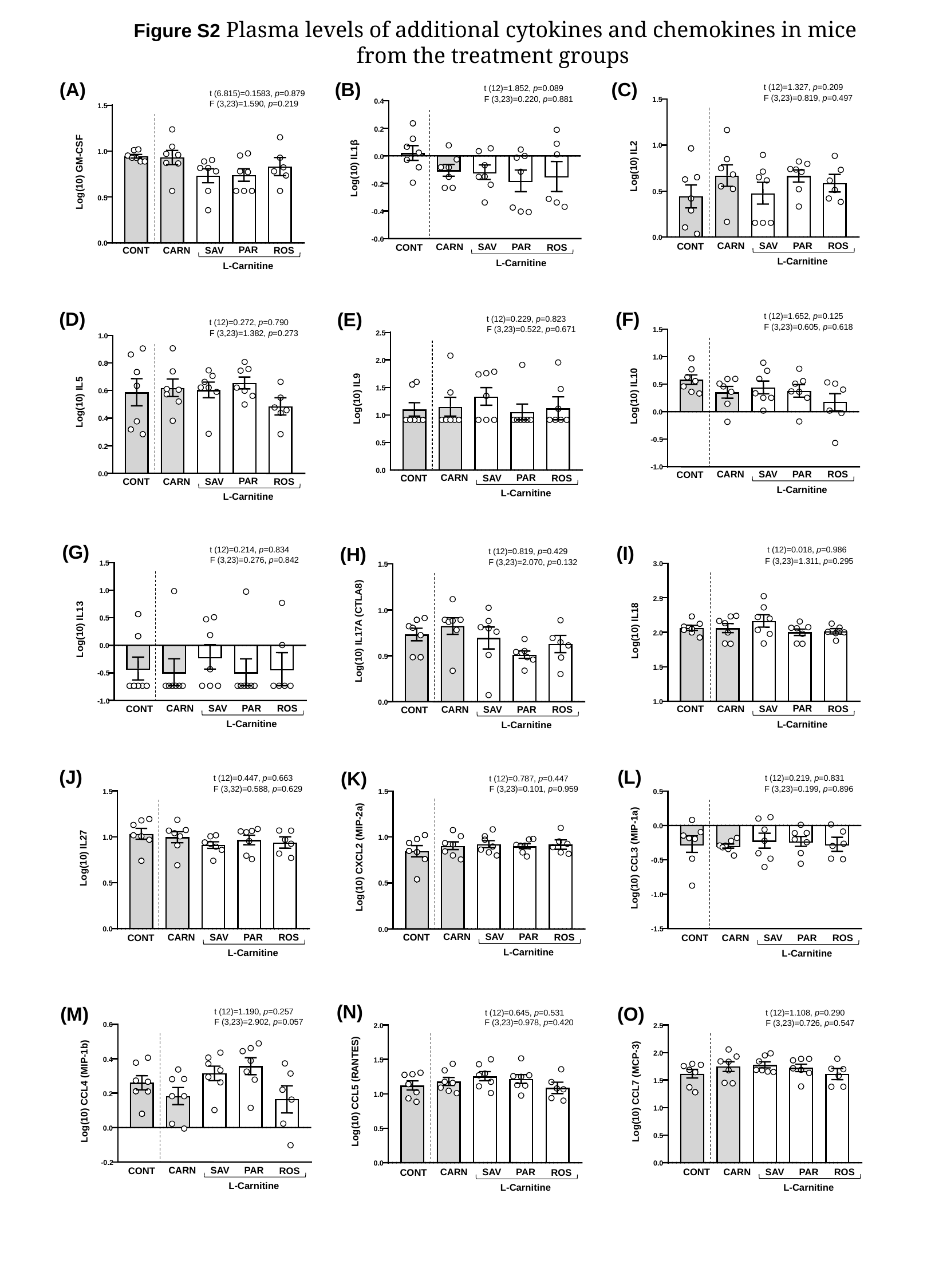

Figure S2 Plasma levels of additional cytokines and chemokines in mice from the treatment groups
(A)
(C)
(B)
t (12)=1.327, p=0.209
t (12)=1.852, p=0.089
t (6.815)=0.1583, p=0.879
F (3,23)=0.819, p=0.497
F (3,23)=0.220, p=0.881
F (3,23)=1.590, p=0.219
1.5
0.4
1.5
0.2
1.0
1.0
0.0
Log(10) IL2
Log(10) IL1β
Log(10) GM-CSF
-0.2
0.5
0.5
-0.4
0.0
-0.6
0.0
PAR
CARN
SAV
ROS
CONT
L-Carnitine
PAR
CARN
SAV
ROS
CONT
L-Carnitine
PAR
CARN
SAV
ROS
CONT
L-Carnitine
(F)
(D)
(E)
t (12)=1.652, p=0.125
t (12)=0.229, p=0.823
F (3,23)=0.522, p=0.671
2.5
2.0
1.5
Log(10) IL9
1.0
0.5
0.0
PAR
CARN
SAV
ROS
CONT
L-Carnitine
t (12)=0.272, p=0.790
F (3,23)=0.605, p=0.618
F (3,23)=1.382, p=0.273
1.5
1.0
1.0
0.8
0.5
0.6
Log(10) IL10
Log(10) IL5
0.0
0.4
-0.5
0.2
-1.0
PAR
CARN
SAV
ROS
CONT
L-Carnitine
0.0
PAR
CARN
SAV
ROS
CONT
L-Carnitine
(G)
(I)
t (12)=0.018, p=0.986
F (3,23)=1.311, p=0.295
3.0
2.5
Log(10) IL18
2.0
1.5
1.0
PAR
CARN
SAV
ROS
CONT
L-Carnitine
(H)
t (12)=0.214, p=0.834
t (12)=0.819, p=0.429
F (3,23)=0.276, p=0.842
F (3,23)=2.070, p=0.132
1.5
1.5
1.0
1.0
0.5
Log(10) IL13
Log(10) IL17A (CTLA8)
0.0
0.5
-0.5
-1.0
0.0
PAR
CARN
SAV
ROS
CONT
L-Carnitine
PAR
CARN
SAV
ROS
CONT
L-Carnitine
(J)
(L)
(K)
t (12)=0.219, p=0.831
t (12)=0.447, p=0.663
t (12)=0.787, p=0.447
F (3,23)=0.199, p=0.896
0.5
0.0
Log(10) CCL3 (MIP-1a)
-0.5
-1.0
-1.5
PAR
CARN
SAV
ROS
CONT
L-Carnitine
F (3,32)=0.588, p=0.629
F (3,23)=0.101, p=0.959
1.5
1.5
1.0
1.0
Log(10) CXCL2 (MIP-2a)
Log(10) IL27
0.5
0.5
0.0
0.0
PAR
CARN
SAV
ROS
CONT
PAR
CARN
SAV
ROS
CONT
L-Carnitine
L-Carnitine
(N)
t (12)=0.645, p=0.531
2.0
1.5
Log(10) CCL5 (RANTES)
1.0
0.5
0.0
PAR
CARN
SAV
ROS
CONT
L-Carnitine
(O)
(M)
t (12)=1.190, p=0.257
t (12)=1.108, p=0.290
F (3,23)=2.902, p=0.057
F (3,23)=0.978, p=0.420
F (3,23)=0.726, p=0.547
0.6
2.5
2.0
0.4
1.5
Log(10) CCL4 (MIP-1b)
Log(10) CCL7 (MCP-3)
0.2
1.0
0.0
0.5
-0.2
0.0
PAR
CARN
SAV
ROS
CONT
PAR
CARN
SAV
ROS
CONT
L-Carnitine
L-Carnitine
