# Supplementary material for: Beneficial Effects of Essential Oils from the Mediterranean Diet on Gut Microbiota and Their Metabolites in Ischemic Heart Disease and Type-2 Diabetes Mellitus
Source: Nutrients. 2022 Nov 3;14(21):4650. doi: 10.3390/nu14214650 (PMC9657080; doi:10.3390/nu14214650)
Supplement: Supplementary file 1 [file nutrients-14-04650-s001.zip › Table S1.pdf]

Table S1. Experimental diet used in animals

Crude Nutrients	%	Additives	Per kg
Crude Protein	22.0	Vitamin A [IU]	15,000
Crude fat	23.6	Vitamin D_3 [IU]	1,500
Crude fibre	5.7	Vitamin E [mg]	150
Crude ash	5.4	Vitamin C [mg]	30
Starch	6.8		
Sugar	21.1	Copper [mg]	12
Energy: 19.3 MJ [or kcal] ME/kg			
