# Supplementary material for: Beneficial Effects of Essential Oils from the Mediterranean Diet on Gut Microbiota and Their Metabolites in Ischemic Heart Disease and Type-2 Diabetes Mellitus
Source: Nutrients. 2022 Nov 3;14(21):4650. doi: 10.3390/nu14214650 (PMC9657080; doi:10.3390/nu14214650)
Supplement: Supplementary file 1 [file nutrients-14-04650-s001.zip › Table S2.pdf]

Table S2. Correlation analysis between gut microbial abundance and plasma levels of cytokines and chemokines

	Phylum			Family			Genus		
	**Name**	**rho**	***p*-value**	**Name**	**rho**	***p*-value**	**Name**	**rho**	***p*-value**
**IL1ß (pg/mL)**				Oxalobacteraceae	-0.455	0.009*			
**IL5 (pg/mL)**							*Desulfovibrio*	+0.376	0.034
**IL6 (pg/mL)**				Alcaligenaceae	+0.418	0.017	*Desulfovibrio*<br>*Pigmentiphaga*	+0.364<br>+0.383	0.041<br>0.030
**IL9 (pg/mL)**	Firmicutes	-0.399	0.024	Lachnospiraceae	-0.350	0.049	*Oscillospira*	-0.381	0.032
**IL10 (pg/mL)**							*Sutterella*	-0.383	0.031
**IL13 (pg/mL)**				Barnesiellaceae	-0.363	0.041	*Bernesiella*	-0.363	0.041
**IL17A (CTLA8) (pg/mL)**				Paraprevotellaceae	+0.401	0.023	*Paraprevotella*	+0.401	0.023
**IL18 (pg/mL)**	Firmicutes	-0.407	0.021	Turicibacteraceae	-0.415	0.018	*Turicibacter*	-0.415	0.018
**IL22 (pg/mL)**							*Clostridium*	+0.467	0.007*
**IL23 (pg/mL)**				Oxalobacteraceae	+0.378	0.033			
**TNFa (pg/mL)**				Rikenellaceae	-0.363	0.041	*Alistipes*	-0.380	0.032
**GM-CSF (pg/mL)**				Lactobacillaceae	-0.526	0.002*	*Lactobacillus*	-0.526	0.002*
**MIP2a (CXCL2) (pg/mL)**	Firmicutes	-0.427	0.015	Ruminococcaceae<br>Desulfovibrionaceae	-0.461<br>-0.371	0.008*<br>0.031	*Oscillospira*<br>*Bilophila*	-0.464<br>-0.458	0.007*<br>0.008*
**IP10 (CXCL10) (pg/mL)**	Tenericutes	-0.376	0.034	Anaeroplasmataceae	-0.376	0.034	*Oscillospira*<br>*Anaeroplasma*	-0.351<br>-0.376	0.049<br>0.034
**MIP1b (CCL4) (pg/mL)**				Rikenellaceae	-0.366	0.039			
**MCP3 (CCL7) (pg/mL)**	Firmicutes	-0.450	0.010	Ruminococcaceae	-0.430	0.014	*Desulfovibrio*<br>*Oscillospira*<br>*Bilophila*	-0.361<br>-0.492<br>-0.441	0.042<br>0.004*<br>0.012

Abbreviations: rho, Spearman correlation coefficient.
P-values were adjusted for multiple testing correlations: (*) denotes significant adjusted p<0.05.
