# Supplementary material for: Beneficial Effects of Essential Oils from the Mediterranean Diet on Gut Microbiota and Their Metabolites in Ischemic Heart Disease and Type-2 Diabetes Mellitus
Source: Nutrients. 2022 Nov 3;14(21):4650. doi: 10.3390/nu14214650 (PMC9657080; doi:10.3390/nu14214650)
Supplement: Supplementary file 1 [file nutrients-14-04650-s001.zip › Table S3.pdf]

Table S3. Correlation analysis between gut microbial abundance and plasma levels of AAS, GGS and pentosidine

	**AAS**		**GGS**		**Pentosidine**	
	**r**	***p*-value**	**r**	***p*-value**	**r**	***p*-value**
**AAS**	1	---	+0.250	0.154	+0.352	0.041
**GGS**	+0.250	0.154	1	---	-0.014	0.936
**Pentosidine**	+0.352	0.041	-0.014	0.936	1	---
**Phylum**	**rho**	***p*-value**	**rho**	***p*-value**	**rho**	***p*-value**
Bacteroidetes	-0.270	0.129	-0.367	0.035	-0.096	0.596
Firmicutes	-0.440	0.010	-0.397	0.022	-0.068	0.705
Lentisphaerae	-0.153	0.394	-0.269	0.131	-0.381	0.029
Proteobacteria	-0.362	0.038	-0.208	0.246	-0.068	0.707
**Family**	**rho**	***p*-value**	**rho**	***p*-value**	**rho**	***p*-value**
Bacteroidacea	-0.386	0.026	-0.370	0.034	-0.206	0.250
Porphyromonadaceae	-0.330	0.061	-0.412	0.017	-0.377	0.031
Barnesiellaceae	-0.155	0.389	-0.558	0.001*	-0.018	0.922
Odoribacteraceae	-0.081	0.655	-0.388	0.026	-0.118	0.512
Paraprevotellaceae	-0.465	0.006*	-0.078	0.665	-0.235	0.188
Lactobacillaceae	-0.271	0.127	-0.499	0.003*	-0.336	0.056
Ruminococcaceae	-0.378	0.030	-0.104	0.566	-0.023	0.898
Victivallaceae	-0.153	0.394	-0.269	0.131	-0.381	0.029
Alcaligenaceae	-0.418	0.016	-0.183	0.309	-0.284	0.109
**Genus**	**rho**	***p*-value**	**rho**	***p*-value**	**rho**	***p*-value**
Bacteroides	-0.386	0.026	-0.370	0.034	-0.206	0.250
Parabacteroides	-0.330	0.061	-0.412	0.017	-0.377	0.031
Barnesiella	-0.150	0.405	-0.560	0.001*	-0.007	0.970
Butyricimonas	-0.081	0.655	-0.388	0.026	-0.118	0.512
Paraprevotella	-0.465	0.006*	-0.078	0.665	-0.235	0.188
Lactobacillus	-0.271	0.127	-0.499	0.003*	-0.336	0.056
Victivallis	-0.153	0.394	-0.269	0.131	-0.381	0.029
Pigmentiphaga	-0.379	0.030	-0.192	0.284	-0.259	0.146

Abbreviations: AAS, alpha-aminoadipic semialdehyde; GGs, gamma-glutamic semialdehyde; r, Pearson correlation coefficient; rho, Spearman correlation coefficient. *P*-values were adjusted for multiple testing correlations: (*) denotes significant adjusted $p<0.05$.
